# Development and Characterization of Poly(1-vinylpyrrolidone-co-vinyl acetate) Copolymer Based Polymer Electrolytes 

**DOI:** 10.1155/2014/254215

**Published:** 2014-11-05

**Authors:** Nurul Nadiah Sa'adun, Ramesh Subramaniam, Ramesh Kasi

**Affiliations:** Centre for Ionics University of Malaya, Department of Physics, Faculty of Science, University of Malaya, 50603 Kuala Lumpur, Malaysia

## Abstract

Gel polymer electrolytes (GPEs) are developed using poly(1-vinylpyrrolidone-co-vinyl acetate) [P(VP-co-VAc)] as the host polymer, lithium bis(trifluoromethane) sulfonimide [LiTFSI] as the lithium salt and ionic liquid, and 1-ethyl-3-methylimidazolium bis(trifluoromethylsulfonyl) imide [EMImTFSI] by using solution casting technique. The effect of ionic liquid on ionic conductivity is studied and the optimum ionic conductivity at room temperature is found to be 2.14 × 10^−6^ S cm^−1^ for sample containing 25 wt% of EMImTFSI. The temperature dependence of ionic conductivity from 303 K to 353 K exhibits Arrhenius plot behaviour. The thermal stability of the polymer electrolyte system is studied by using thermogravimetric analysis (TGA) while the structural and morphological properties of the polymer electrolyte is studied by using Fourier transform infrared (FTIR) spectroscopy and X-ray diffraction analysis (XRD), respectively.

## 1. Introduction

Polymer electrolytes are gaining great interest due to their high ionic conductivity and wide electrochemical windows. They also possessed interesting properties such as thin-film forming ability, flexibility, and transparency [1–3]. There are three types of polymer electrolytes: (i) solid polymer electrolytes (SPEs), (ii) gel polymer electrolytes (GPEs), and (iii) composite polymer electrolytes [4]. Among the three stages, GPEs possessed the best properties such as increase in safety performance by preventing leakage in the polymer matrix, decrease in reactivity, and significant improvement in ionic conductivity with a small amount of plasticizers [5]. GPEs also exhibit both diffusive property of liquids and cohesive property of solids [4].

Ionic liquids (ILs) are also known as “green solvents” because of their disability to evaporate into the air as they do not have measurable vapor pressure [6]. ILs have been widely used in various fields of chemistry and industry due to their chemical and thermal stability, low vapor pressure, and high ionic conductivity properties [7]. Fuller et al. successfully prepared poly(vinylidene fluoride)-hexafluoropropylene copolymer [PVdF(HFP)] based gel polymer electrolytes with ionic liquids, 1-ethyl-3-methylimidazolium salts of triflate (CF_3_SO_3_^−^) and BF_4_^−^. The obtained films are freestanding and flexible with ionic conductivities ranging from 1.1 to 5.8 mS cm^−1^ at room temperature [8]. Tiyapiboonchaiya et al. had done a research on polymer-in-ionic-liquid electrolyte in order to increase the ionic conductivity of polymer electrolytes. They were using poly(1-vinylpyrrolidone-co-vinyl acetate) [P(VP-co-VAc)] copolymer in 1-ethyl-3-methylimidazolium bis(trifluoromethylsulfonyl) imide (EMImTFSI) resulting in a gel polymer electrolyte. For copolymer concentrations up to 30 wt.%, the ionic conductivity is found to be around 10^−3^ S cm^−1^ at 22°C [9].

In the present study, the effect of ionic liquid, EMImTFSI, on the ionic conductivity of poly(1-vinylpyrrolidone-co-vinyl acetate) [P(VP-co-VAc)]/lithium bis(trifluoromethane) sulfonimide [LiTFSI] polymer electrolytes system is investigated using ac impedance spectroscopy at different temperatures. In addition, the thermal stability, structural and morphological properties of the polymer electrolyte system is analysed using thermogravimetric analysis (TGA), Fourier transform infrared (FTIR) spectroscopy, and X-ray diffraction analysis (XRD), respectively.

## 2. Experimental

The materials used in this research work are P(VP-co-VAc) with a molecular weight of ~5.0 × 10^4^ g mol^−1^ (Sigma-Aldrich), lithium salt LiTFSI with 97% purity (Sigma-Aldrich), and ionic liquid EMImTFSI with 98% purity (Basionics) which were used without further purification in this study. The gel polymer electrolytes were prepared by using solution casting technique using distilled water as a solvent.

Table 1 shows the compositions and designation of produced polymer electrolytes. The ratio of copolymer to lithium salt is fixed at 70 : 30 in weight percentage (wt%). Suitable amounts of P(VP-co-VAc), LiTFSI, and EMImTFSI were dissolved in distilled water. The solution was stirred at room temperature continuously for 24 hours in order to obtain a homogeneous solution. Then, the solution was cast in a glass petri dish and left to dry slowly in the oven at 60°C to produce mechanically stable gel polymer electrolytes. The samples were prepared until the corporation of 25 wt% of EMImTFSI as further addition of EMImTFSI would be resulting in mechanically unstable gel polymer electrolyte films.

The casted thin films were then characterized on its electrical, thermal, structural, and morphological properties.

*Electrical Properties: Ac Impedance Spectroscopy.* HIOKI 3532-50 LCR HiTESTER was used to measure the impedance of the samples. The thickness of the samples was measured using a micrometer screw gauge. The impedance measurement for each sample was done in the frequency range from 50 Hz to 5 MHz at room temperature to 80°C. The samples were placed on the sample holder in between two stainless steel electrodes with diameter of 4.9087 cm^2^. Ionic conductivity, *σ*, was determined from the following equation:

(1) $$\begin{array}{c} \sigma =\frac{L}{R_{b}A}, \end{array}$$

where *L* is the sample thickness (cm), *A* is the cross-sectional area of electrode and sample contact (cm^2^), and *R*_*b*_ is the bulk resistance (Ω) of the sample.

*Thermal Properties: Thermogravimetric Analysis (TGA).* TA TGA Q500 was used to study the thermal stability of the samples. The samples were cut and weighed ~2.0 mg. Then, the samples were put into the sample holder and heated under nitrogen atmosphere from 25°C to 505°C at a heating rate of 10°C min^−1^.

*Structural Properties: Fourier Transform Infrared (FTIR) Spectroscopy.* The FTIR studies were carried out by using Thermo Scientific Nicolet iS10 spectrophotometer at room temperature in the wave region between 4000 cm^−1^ and 600 cm^−1^ with a resolution of 4 cm^−1^. 

*Morphological Properties: X-Ray Diffraction Analysis (XRD).* The XRD study was conducted by using D5000 diffractometer to determine the polymer electrolytes films which are either crystalline or amorphous in nature based on the characteristic pattern obtained from X-ray diffractogram. Coherent length study is done to justify that the crystallinity of P(VP-co-PVAc)—LiTFSI—EMImTFSI system is reduced. The coherent length, *C*, is calculated from the Scherrer equation:

(2) $$\begin{array}{c} C=\frac{0.9\lambda }{\cos\theta_{b}\left(\Delta 2\theta_{b}\right)}, \end{array}$$

where *λ* is the X-ray wavelength; *θ*_*b*_ is the glancing angle; and Δ2*θ*_*b*_ is the full width at half maximum (FWHM).

## 3. Results

### 3.1. Electrical Properties

#### 3.1.1. Ionic Conductivity Studies at Room Temperature

**Figure 1:**
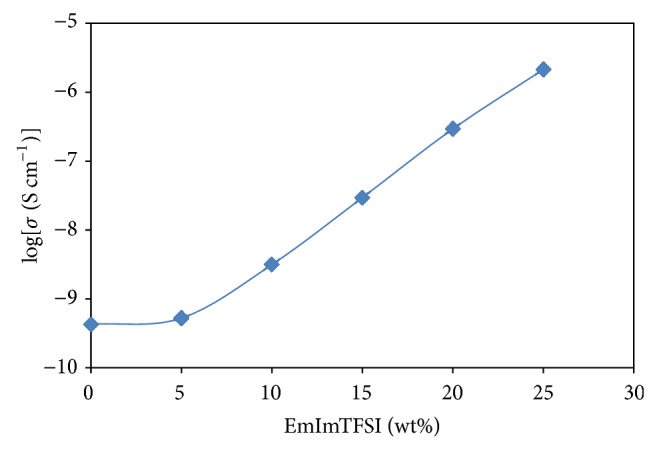
The variation of ionic conductivity with different wt% of EMImTFSI at room temperature.

Figure 1 shows the variation of ionic conductivity of P(VP-co-VAc)/LiTFSI polymer electrolytes system with the incorporation of EMImTFSI at room temperature. From Figure 1, it can be observed that the ionic conductivity for EMIm 0 is 4.27 × 10^−10^ S cm^−1^. The maximum ionic conductivity 2.14 × 10^−6^ S cm^−1^ is achieved with addition of 25 wt% of EMImTFSI to the polymer electrolyte system.

#### 3.1.2. Temperature Dependent Ionic Conductivity Studies

**Figure 2:**
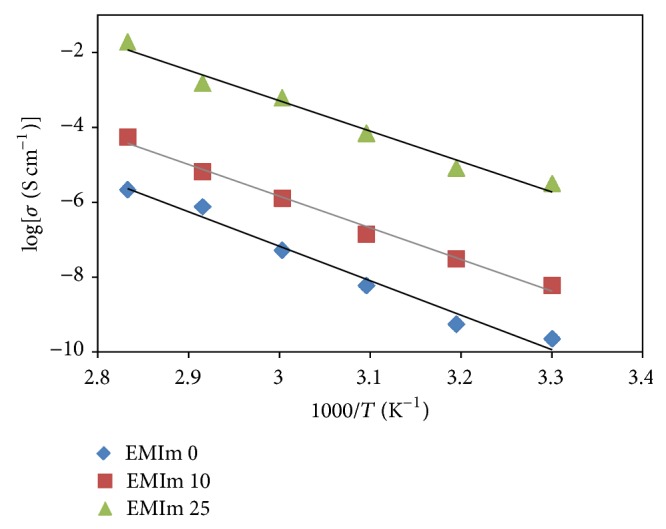
Arrhenius plots of ionic conductivity of EMIm 0, EMIm 10, and EMIm 25.

Plots of linear variation of log ionic conductivity (*σ*) against 1000/*T* (in Kelvin) are shown in Figure 2. It can be observed that the polymer electrolytes system obeys the Arrhenius rule. Activation energy, *E*_*a*_, for each system can be determined from the slope of its Arrhenius plot.  Table 2 shows the values of *E*_*a*_ for the polymer electrolytes.

### 3.2. Thermal Properties

#### 3.2.1. Thermogravimetric Analysis (TGA)

**Figure 3:**
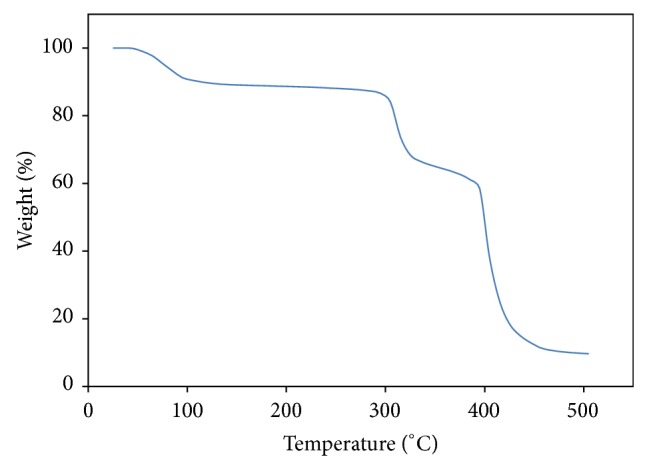
Thermogravimetric curve of pure P(VP-co-VAc).

**Figure 4:**
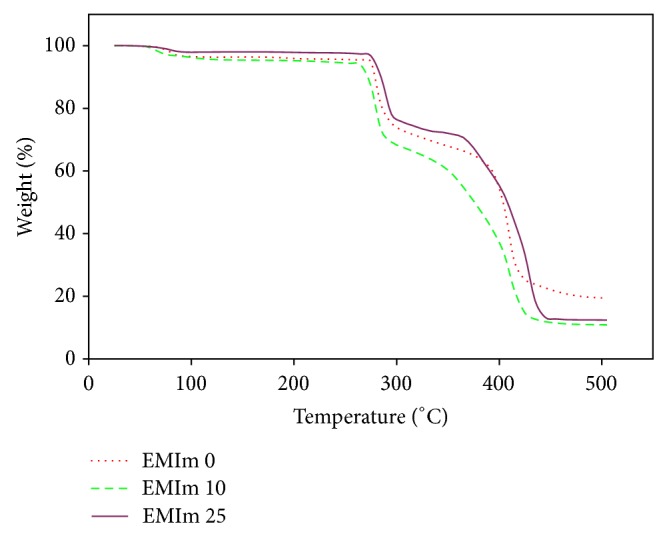
Thermogravimetric curves of EMIm 0, EMIm 10, and EMIm 25.

Figures 3 and 4 show the thermogravimetric curves for pure P(VP-co-VAc), EMIm 0, EMIm 10, and EMIm 25. From these figures, it can be observed that all degradation curves experience three degradation stages. There is a small weight loss at temperature below 100°C followed by a stable weight. Two main degradation stages are at higher temperature range.

### 3.3. Structural Properties

#### 3.3.1. Fourier Transform Infrared (FTIR) Spectroscopy

**Figure 5:**
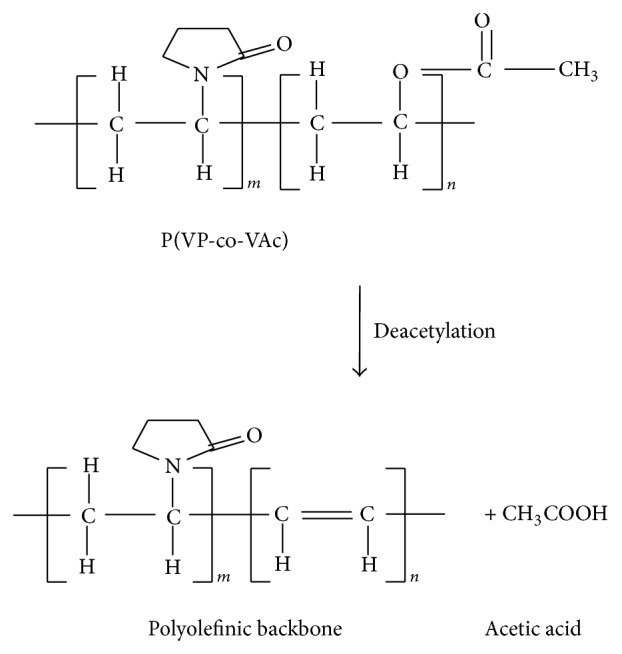
Deacetylation of PVAc—the second degradation stage of P(VP-co-Vac).

**Figure 6:**
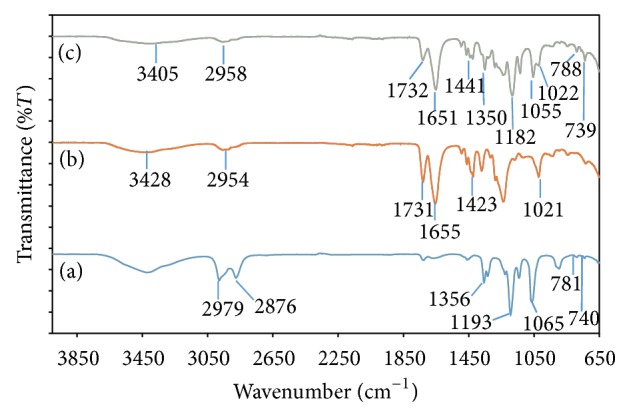
FTIR spectra for (a) pure LiTFSI, (b) pure P(VP-co-VAc), and (c) EMIm 0.

Figure 5 shows the FTIR spectra for P(VP-co-VAc)-LiTFSI system while Figure 6 represents the changes in characteristic peaks of P(VP-co-VAc)-LiTFSI system with addition of ionic liquid, EMImTFSI. Based on both figures, we can observe that there are apparent changes occurring to the characteristic peaks of the polymer systems, which confirmed the cooperative interactions between P(VP-co-VAc), LiTFSI, and EMImTFSI.

### 3.4. Morphological Properties

#### 3.4.1. X-Ray Diffraction Analysis (XRD)

Figure 8 depicts the X-ray diffractogram of pure P(VP-co-VAc), pure LiTFSI, EMIm 0, EMIm 10, and EMIm 25 based complexes. It can be observed in Figure 8(i) that the XRD pattern of pure LiTFSI shows intense peaks at Bragg angles of 13.6°, 15.9°, 18.6°, 18.9°, and 21.4°, which reveal the crystalline nature of the lithium salt. Figure 8(ii) shows two amorphous humps at angles of 2*θ* = 15.4° and 19.5°, revealing the semicrystalline nature of pure P(VP-co-VAc). The addition of LiTFSI into the polymer system shows that the diffraction peaks corresponding to the salt are disappearing, suggesting that LiTFSI is fully complexed with P(VP-co-VAc).

The diffractograms of P(VP-co-VAc)-LiTFSI with different wt% of EMImTFSI are shown in Figures 8(iv) and 8(v). It can be observed that the amorphous humps present in the EMIm25 system are broader than the humps in EMIm 10. The intensity of the humps in EMIm 25 is also lower than the intensity of humps in EMIm 10.

Table 2 shows the value of the coherent length for the polymer electrolytes system.

## 4. Discussion

### 4.1. Electrical Properties

#### 4.1.1. Ionic Conductivity Studies at Room Temperature

Incorporation of ionic liquid, EMImTFSI, to the polymer electrolyte gives plasticizing effect which increases the flexibility of the polymer backbone and enhances the amorphous phase of the system. In addition, both Im^+^ and TFSI^−^ ions in the ionic liquid are mobile, thus increasing the number of charge carriers available for conduction. Besides, with the low viscosity of EMImTFSI, the crystallinity of the polymer matrix can be reduced, hence increasing the mobility of the charge carriers [10]. The increasing in ionic conductivity is also due to high self-dissociating and ion-transporting abilities of EMImTFSI [3].

The increase in ionic conductivity as a function of temperature is due to the decrease in viscosity of the polymer systems that increases the chain flexibility [11]. This phenomenon can be said to be the ions hopping mechanism between coordinating sites, local structural relaxations, and segmental motions of the polymer system [12].

#### 4.1.2. Temperature Dependent Ionic Conductivity Studies

From Figure 2, since the polymer electrolytes system obeys the Arrhenius rule, the conductivity of the system can be expressed as

(3) $$\begin{array}{c} \sigma =\;\sigma_{o}\exp\left(\frac{{-E}_{a}}{kT}\right), \end{array}$$

where *σ*_*o*_ is the preexponential factor; *E*_*a*_ is the activation energy; *k* is the Boltzmann constant; and *T* is the absolute temperature.

Activation energy, *E*_*a*_, can be defined as the energy needed to overcome the reorganization and reformation of Li^+^ to relocate to neighbouring sites. From Table 2, it can be seen that the activation energy values are decreasing as the value of ionic conductivity of the polymer electrolytes systems increased. The decreasing of activation energy suggests that the amorphous nature of the polymer electrolytes enables the Li^+^ to move faster inside the polymer network. Upon addition of EMImTFSI, the interaction between the polymer system and lithium ions is disturbed causing less energy which is needed for the Li^+^ hopping process [13].

### 4.2. Thermal Properties

#### 4.2.1. Thermogravimetric Analysis (TGA)

There are three degradation stages which can be observed from Figures 3 and 4. The first degradation stage for all samples occurred at temperature below 100°C with percentage weight loss of 10% in pure polymer system, 3% in EMIm 0, 4% in EMIm 10, and 2% in EMIm 25. This is due to the removal of water adsorbed caused by the hydrophilicity of P(VP-co-Vac) [14] and hygroscopic nature of LiTFSI. Volatilization of the monomers and oligomers adsorbed in the polymer system [15] is also contributed to the first degradation stage. Amount of water adsorbed by the polymer electrolytes is reduced with the increasing concentration of EMImTFSI due to its hydrophobic nature.

For the second degradation stage, it is shown that the pure polymer is starting to degrade at 295°C with 24% weight loss. In EMIm 0, the second degradation stage occurred at 275°C with 30% weight loss while in EMIm 10 and EMIm 25 it occurred at temperature 265°C with weight loss of 30% and 275°C with 25% weight loss, respectively. This stage corresponds to the degradation of poly(vinyl acetate) (PVAc) due to the total loss of acetic acid as shown in Figure 5 [16].

The third stage of degradation happened due to the degradation of vinylpyrrolidone [14] and vinyl acetate [16]. The degradation of vinylpyrrolidone in this stage involved the scission of N–C=O to form ester and the release of ammonia gas, NH_3_ [14]. The polyolefinic backbone of vinyl acetate is further degraded to benzene, toluene, and naphthalene. At this temperature range, the C=C in the polyolefinic backbone are favourable to* cis*-trans isomerisation, aromatization, and cross-linking [16]. These results show that vinylpyrrolidone is more thermally stable than vinyl acetate [17]. For the pure polymer system, the third degradation stage occurred at temperature 385°C with percentage weight loss of 51% and at 385°C with weight loss of 43% in EMIm 0. For EMIm 10 system, the third degradation stage occurred at temperature 345°C with weight loss of 59% and at temperature 365°C with 58% weight loss for the EMIm 25 system.

As EMImTFSI is nonflammable and nonvolatile, these properties contribute to the increase in heat resistivity of the samples resulting in EMIm 25 to have less weight loss compared to pure P(VP-co-VAc) with residual mass of 12.44% and 9.66%, respectively. Therefore, it can be concluded that samples with addition of EMImTFSI are more thermally stable than pure P(VP-co-VAc).

### 4.3. Structural Properties

#### 4.3.1. Fourier Transform Infrared (FTIR) Spectroscopy

Figure 6 shows the FTIR spectra for P(VP-co-VAc)-LiTFSI system. Peak at 3428 cm^−1^ and 2954 cm^−1^ which is assigned to O–H stretching and C–H stretching of P(VP-co-VAc), respectively, is observed to be shifted to 3405 cm^−1^ and 2958 cm^−1^, respectively, in EMIm 0. It is also observed that the peaks of C=O stretching of both PVAc and PVP at 1731 cm^−1^ and 1655 cm^−1^ have shifted to 1732 cm^−1^ and 1651 cm^−1^, respectively, in the PE system. These shifting might be due to the interaction between free Li^+^ with the ester C=O in PVAc and amide C=O in PVP. Besides, the peak shift of C=O in PVP (Δ*f* = 4 cm^−1^) is more intensive as compared with PVAc (Δ*f* = 1 cm^−1^); therefore it is claimed that interaction of Li^+^ with C=O of PVP is more favourable [18].

From the figure, we can also observe the shifting in the LiTFSI characteristic peaks. The peaks of LiTFSI at 740 cm^−1^ (vibrational (S–N)), 781 cm^−1^ (vibrational (C–S) + vibrational (S–N)), 1193 cm^−1^ (vibrational (CF_3_)_s_), and 1356 cm^−1^ (vibrational (SO3)_a_) are shifted to 739 cm^−1^, 788 cm^−1^, 1182 cm^−1^, and 1350 cm^−1^ in the EMIm 0, respectively. These shifting can be attributed by the presence of free TFSI^−^ in the EMIm 0. Two characteristic peaks of LiTFSI *δ*CH_3_ at 2979 cm^−1^ and 2876 cm^−1^ have disappeared after interacting with P(VP-co-VAc) and C–H stretching peaks of P(VP-co-VAc) at 2954 cm^−1^ shifted to 2958 cm^−1^ in EMIm 0. Besides, the characteristic peak at 1423 cm^−1^ in P(VP-co-VAc) has shifted to 1441 cm^−1^ in EMIm 0. Characteristic peak at 1021 cm^−1^ in P(VP-co-VAc) has also shifted to wavenumber 1022 cm^−1^ and becomes the shoulder peak to LiTFSI characteristic peak that shifted from wavenumber 1065 cm^−1^ in LiTFSI to 1055 cm^−1^ in EMIm 0.

**Figure 7:**
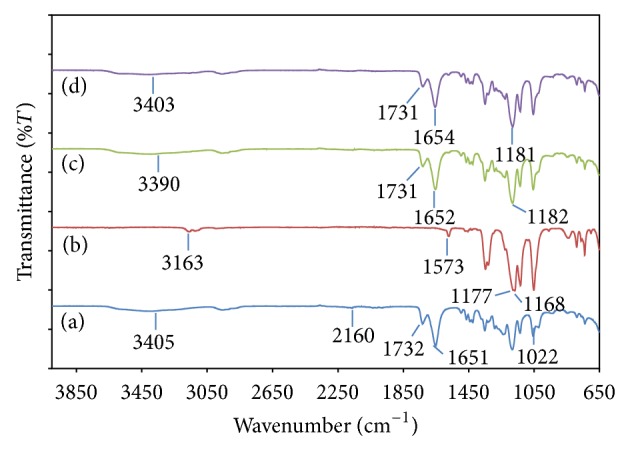
FTIR spectra for (a) EMIm 0, (b) pure EMIm TFSI, (c) EMIm 10, and (d) EMIm 25.

Figure 7 represents the changes in characteristic peaks of P(VP-co-VAc)-LiTFSI system with addition of ionic liquid, EMIm TFSI. The O–H stretching band at 3405 cm^−1^ in the EMIm 0 is observed to be shifted to 3390 cm^−1^ and 3403 cm^−1^ in EMIm 10 and EMIm 25 systems, respectively. The C=O stretching of PVAc and PVP in the EMIm 0 system is also shifted from 1732 cm^−1^ and 1651 cm^−1^, respectively, to 1731 cm^−1^ and 1652 cm^−1^ in EMIm 10 and to 1731 cm^−1^ and 1654 cm^−1^ in EMIm 25.

Upon adding EMImTFSI into the polymer electrolyte system, there are no significant changes occurring to LiTFSI characteristic peaks except for the peak of wavenumber 1022 cm^−1^ which shifted to a shoulder peak in EMIm 10 and disappears in EMIm 25.

It can also be observed from Figure 7(b) that the characteristic peaks of EMImTFSI at 3163 cm^−1^ and 1573 cm^−1^ have disappeared after being added to EMIm 0 system as shown in Figures 7(c) and 7(d). In addition, peak at wavenumber 1177 cm^−1^ and 1168 cm^−1^ in Figure 7(b) is observed to be combined and become peak at 1182 cm^−1^ in Figure 7(c) and 1181 cm^−1^ in Figure 7(d).

### 4.4. Morphological Properties

#### 4.4.1. X-Ray Diffraction Analysis (XRD)

**Figure 8:**
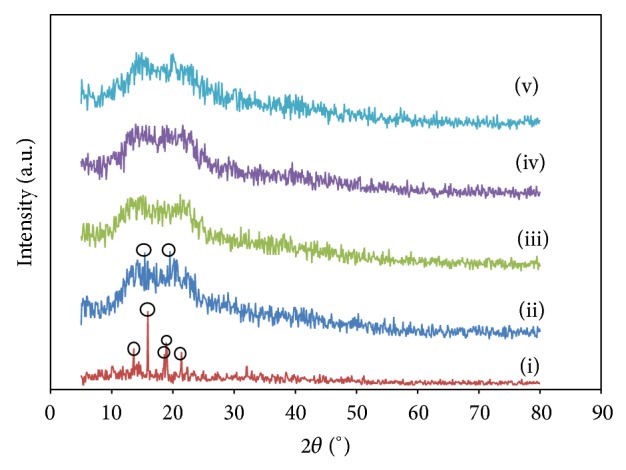
XRD pattern of (i) pure LiTFSI, (ii) pure P(VP-co-VAc), (iii) EMIm 0, (iv) EMIm 10, and (v) EMIm 25.

As shown in Figure 8(iii), the diffraction peaks corresponding to the salt are decreasing with the addition of LiTFSI into the polymer system. These observations indicate that LiTFSI is fully complexed with P(VP-co-VAc) and that the polymer electrolyte has achieved complete dissolution [19]. PVP is capable of forming complexation with various types of lithium salt [20] while PVAc can effectively solvate the lithium cations in order to form homogeneous solution [13].

Based on Figures 8(iv) and 8(v), the increasing size of the amorphous humps in EMIm 25 system and the decreasing intensity of the humps indicate that the addition of EMImTFSI further increases the amorphous nature of the polymer system and leads to higher conductivity. The increase in the amorphous nature induces irregular arrangement molecules in the polymer system; hence a more flexible polymer chain is formed [12, 19].

In general, coherent length defines the crystallite size. As the diffraction peak width is longer, the crystallite size is shorter and thus brings higher amorphous phase and lower crystallinity of the polymer system [21]. Based on Table 2, it is further proved that EMIm 25 is more amorphous than EMIm 10 as EMIm 25 has shorter coherent length value than EMIm 10.

## 5. Conclusion

In this work, GPEs are developed by incorporating different amount of EMImTFSI into the polymer electrolytes system. Among all the polymer electrolytes systems, EMIm 25 has the highest ionic conductivity, 2.14 × 10^−6^ S cm^−1^, which suggested that addition of ionic liquid in the polymer electrolytes systems increases the ionic conductivity values. The temperature dependence ionic conductivity study shows that all samples exhibit Arrhenius type which is favourable for Li^+^ ion hopping mechanism at higher temperature as proved by the increasing in ionic conductivity values with temperature. In addition, the addition of EMImTFSI also enhanced the thermal stability of the polymer electrolytes systems. The FTIR and XRD studies showed the complexation of the materials inside the polymer electrolytes systems which revealed that with the incorporation of EMImTFSI the amorphous fraction is increased, resulting in the increasing of the ionic conductivity values.

## Figures and Tables

**Table 1:** Designation of the developed polymer electrolytes with its respective materials composition ratio.

Designations	Polymer electrolytes composition (P(VP-co-VAc)/LiTFSI : EmImTFSI) (wt%)
EMIm 0	100 : 0
EMIm 5	95 : 5
EMIm 10	90 : 10
EMIm 15	85 : 15
EMIm 20	80 : 20
EMIm 25	75 : 25

**Table 2:** Activation energy and coherent length of EMIm 0, EMIm 10, and EMIm 25 obtained from temperature dependent ionic conductivity and XRD analysis.

Samples	Activation energy, *E*_*a*_ (eV)	Coherent length, *C* (Å)
EMIm 0	1.055	0.099
EMIm 10	0.967	0.087
EMIm 25	0.928	0.017
